# Difference in macrophage migration inhibitory factor between preterm and term newborns and associating clinical factors: Preliminary study

**DOI:** 10.1097/MD.0000000000030223

**Published:** 2022-08-26

**Authors:** Ji Sook Park, Jin Su Jun, Jae Young Cho, Jung Sook Yeom, Ji-Hyun Seo, Jae Young Lim, Chan-Hoo Park, Hyang-Ok Woo, Hee-Shang Youn

**Affiliations:** a Department of Pediatrics, Gyeongsang National University College of Medicine and Gyeongsang National University Hospital, Jinju, South Korea; b Institute of Health Sciences, Gyeongsang National University, Jinju, South Korea; c Department of Pediatrics, Gyeongsang National University Changwon Hospital, Changwon, South Korea.

**Keywords:** macrophage migration inhibitory factor, necrotizing enterocolitis, newborn, preterm

## Abstract

This study aimed to investigate the macrophage migration inhibitory factor (MIF) and associated clinical factors in neonates. Clinical information and blood samples were obtained from 77 neonates. Clinical details were reviewed from medical records, and MIF was measured by enzyme-linked immunosorbent assay using blood samples acquired within a week after birth. Statistical analyses were performed between plasma MIF concentration and clinical factors. Among the 77 newborn infants, 25 were born at <34 weeks of gestation (preterm), 25 at 34 to 37 weeks (late preterm), and 27 at term gestation. The mean MIF was 9849.5 ± 7187.8 pg/mL in preterm, 5718.7 ± 4596.4 in late preterm, and 5361.1 ± 3895.7 in term infants (*P* = .016). Among 25 preterm infants born at <34 weeks of gestation, MIF was significantly higher in infants with necrotizing enterocolitis (NEC, 19,478.6 ± 8162.4 pg/mL, n = 5) than that in infants without NEC (feeding intolerance 7173.7 ± 4203.0 pg/mL, n = 12 and others 7844.9 ± 5311.2 pg/mL, n = 8, *P* = .020). Elevated plasma MIF levels in the transitional period were significantly associated with preterm birth before 34 weeks of gestation and the development of NEC.

## 1. Introduction

Macrophage migration inhibitory factor (MIF) is a multifaceted inflammatory cytokine that exists in the blood stream at low concentrations (ng/mL) in healthy adults.^[1]^ Preformed MIF is present in the cytoplasm and is rapidly released into the bloodstream in response to microbial and hypoxic stimuli.^[2,3]^ MIF has been studied in the pathogenesis of various diseases such as sepsis, cancer, autoimmune, and metabolic diseases in adults.^[3]^ Previous studies have reported a higher level of plasma MIF in neonates than in children and adults, and a higher level of MIF could play a major role in the innate immune response.^[4]^

However, few neonatal MIF studies have been conducted to date. Preterm newborn infants often have a higher susceptibility to infection than term infants because of the detrimental effects on immature organs and the immune system.^[5,6]^ However, the vulnerability of preterm infants to infection is not confined to an immature immune system, and it is unclear how the set point of the immune system is regulated.^[7]^ Compared with term infants, preterm newborns are susceptible to respiratory distress syndrome (RDS), intraventricular hemorrhage (IVH), necrotizing enterocolitis (NEC), and bronchopulmonary dysplasia (BPD), in addition to being vulnerable to infection. Although immaturity may be the main factor, several inflammatory responses may play a role in the pathogenesis of various diseases in preterm infants. Based on the higher MIF in neonates than in adults and the different neonatal morbidities between preterm and term infants, we hypothesized that MIF might be different between preterm and term infants and contribute to initiating an inflammatory cascade associated with neonatal morbidities in preterm infants. Therefore, we investigated the plasma MIF concentration in the early postnatal period and statistically analyzed associations between MIF and clinical factors in neonates.

## 2. Methods

### 2.1. Subjects and blood samples

To investigate the plasma MIF concentration in the early postnatal period and to analyze an association between MIF and neonatal morbidity, neonates with blood samples collected within 1 week after birth and stored at Biobank of Korea were included. Remnant neonatal blood samples after laboratory examination and matched encoded clinical information have been collected prospectively after parental consent is achieved since 2008 by Gyeonsang National University Hospital (GNUH), a member of Biobank of Korea. We obtained randomly assigned 81 blood samples and matched encoded medical records were obtained from Biobank of Korea, and excluded 3 major congenital anomalies (congenital cyanotic heart disease and 2 congenital intestinal obstructions) and chromosomal abnormality (Down syndrome). The clinical characteristics were reviewed as follows:

1. Obstetric problems, including prolonged rupture of membrane (PROM), maternal hypertensive disease, preterm labor pain, use of prenatal steroids, multiple gestations, and others, were reviewed from medical records under the following definitions: PROM, leakage of amniotic fluid over 18 hours before giving birth, maternal hypertensive disease as underlying essential hypertension, pregnancy-induced hypertension, preeclampsia, eclampsia, or abruption of the placenta caused by hypertension; preterm labor pain as pain caused by uterine contraction that occurred before 37 weeks of gestation and could not be controlled by medication; and prenatal steroid injection of betamethasone to the mother before giving birth.
2. The perinatal clinical factors reviewed included gestational age at birth, birth weight, sex, Apgar score at 5 minutes, and delivery method.
3. Postnatal clinical course or outcomes that were reviewed included RDS, patent ductus arteriosus (PDA), IVH, neonatal jaundice, sepsis, NEC, BPD, feeding formula, hospital stays, and mortality and were defined as follows: RDS as ground glass opacity in both lungs on chest X-ray with respiratory difficulties or desaturation; PDA as an existence of hemodynamically significant shunt of the left to right direction from the aorta to the pulmonary artery with ≥1.4 ratio of the diameter of the left atrium to the aorta on echocardiography; IVH as intraventricular or intracerebral hemorrhage on cranial ultrasonography; neonatal jaundice as an unconjugated hyperbilirubinemia indicated by phototherapy^[8]^; sepsis as positivity of culture studies using blood, urine, cerebrospinal fluid, or other bodily fluids; NEC as stage ≥2 of the modified Bell classification^[9]^; duration of full enteral nutrition (FEN) was investigated and FEN was determined when the infants could achieve an enteral nutrition of ≥100 mL/kg/d; feeding intolerance was determined by gastrointestinal symptoms or signs to delay the progression of enteral nutrition not fulfilled with NEC definition based on modified Bell classification^[9]^; BPD as a pulmonary condition in which supplemental oxygen was necessary at 36 weeks of gestation or postnatal 28 days.

A total of 77 blood samples from neonates in the early postnatal period and medical records of the 77 neonates were divided into 3 groups such as preterm infants born at <34 weeks of gestation, late preterm infants at 34 to 37, and term infants.

Remnant blood samples after laboratory tests for clinical purposes were collected by GNUH with parental consent and stored at −80°C until tested for this study. The 77 neonatal blood samples were taken on the second day after birth (mean 2.9 ± 2.5 days).

### 2.2. Plasma MIF and cytokines

Plasma MIF was measured by enzyme-linked immunosorbent assay (ELISA) using a human MIF ELISA kit (LSBio, Seattle, WA). Concentrations were statistically analyzed according to clinical factors, including gestational age at birth, obstetric problems, and perinatal or postnatal clinical factors. Comparisons of MIF were performed according to each prematurity-related disease (RDS, PDA, IVH, NEC, and BPD) among 25 preterm neonates born at <34 weeks of gestation.

Plasma concentrations of interleukin (IL) 1β, IL6, IL8, IL10, tumor necrosis factor (TNF)-α, and interferon (IFN)-γ were measured by ELISA using a human multi-cytokine ELISA kit (Custom Assay kit; Quansys Biosciences, Logan, UT) and statistically analyzed for correlation with MIF. The limits of detection of IL1β, IL6, IL8, IL10, TNF-α, and IFN-γ were 0.19, 0.051, 0.037, 0.069, 0.032, and 0.12 pg/mL, respectively.

### 2.3. Statistical analyses

Continuous variables were reported as mean and standard deviation, and compared according to clinical factors using the Kruskal-Wallis or Mann–Whitney *U* test. The non-parametric Spearman test was used to assess the correlation between continuous variables. Categorical variables were reported as numbers and percentages, and compared using the chi-square or Fisher exact test. The *P* value was set at <0.05. Statistical analyses were performed using SPSS Statistics (version 25.0; IBM, Armonk, NY), and graphics were created using GraphPad Prism 9 (GraphPad Software, San Diego, CA).

This study was reviewed and approved by the Institutional Review Board of Gyeongsang National University Hospital (GNUH 2018-06-007) and conducted at GNUH, in 2018–2019, retrospectively.

## 3. Results

### 3.1. Clinical characteristics

A total of 77 neonates were born at 35.2 ± 3.0 weeks of gestation and weighed 2318.4 ± 623.5 g. Among them, 25 were born at <34 weeks of gestation, 25 at 34 to 37, and 27 at term. The causes of admission were prematurity (n = 42, 53.8%), small for gestational age (n = 16, 20.5%), and maternal conditions that could affect the baby’s health status, such as gestational diabetes mellitus or thyroid disease (n = 14, 17.9%). Three of 16 small for gestational ages, 4 of 14 neonates admitted due to maternal condition, and 1 with pneumothorax of 5 others were born at <37 weeks of gestation. Two prematurely born infants died at 5 and 12 months after birth because of sepsis and pneumonia, respectively. The clinical characteristics of patients are presented in Table 1. Sex and delivery method were not different between the 3 groups. Apgar score at 5 minutes was lower in preterm neonates born at <34 weeks of gestation than in others. Obstetric problems including PROM and maternal hypertensive disorders were more often in preterm neonates born at <34 weeks of gestation than in others. Multiple gestation was more frequently occurred in both preterm neonates groups (n = 7) than in term (n = 1). Rate of breast feeding was not different between the 3 groups. Neonatal morbidities were more frequently occurred in preterm neonates born at <34 weeks of gestation. Duration for achieving FEN (20.4 ± 19.6 days vs 2.8 ± 1.3 days in preterm neonates born at 34–37 and 2.5 ± 2.3 days in term neonates) and hospital stays (49.1 ± 38.3 days vs 11.8 ± 4.27 days in preterm neonates born at 34–37 and 8.74 ± 8.44 days in term neonates) were longer in preterm neonates born at <34 weeks of gestation than in others.

**Table 1:** Clinical characteristics of 77 neonates.

Variables	Total (n = 77)	Preterm neonate (n = 50)		Term neonate (n = 27)	*P* values
		<34 (n = 25)	34–37 (n = 25)		
Gestation at birth (wk, mean ± SD)	35.2 ± 3.0	31.9 ± 1.58	34.7 ± 0.59	38.6 ± 0.98	<.001
Birth weight (g, mean ± SD)	2318.4 ± 623.5	1715.2 ± 402.2	2305.6 ± 213.5	2888.9 ± 506.8	<.001
Female, n (%)	33 (42.9)	9 (36.0)	11 (44.0)	13 (48.1)	.653
Vaginal delivery, n (%)	27 (35.1)	9 (36.0)	8 (32.0)	10 (37)	.955
5′-AS (mean ± SD)	8.6 ± 1.6	7.9 ± 2.28	9.0 ± 1.14	9.0 ± 0.81	.040
Obstetric problems, n (%)	64 (81.8)	25 (96.0)	23 (60.0)	16 (88.9)	.004
PROM	20	10	10	0	
Maternal hypertensive disease	14	8	5	1	
Maternal gestational diabetes mellitus	11	0	2	9	
Others	19	7	6	6	
Prenatal Steroid, n (%)	27 (35.5)	16 (64.0)	1 (4.0)	10 (38.5)	<0.001
Multiple gestation, n (%)	8 (10.4)	4 (16.0)	3 (12.0)	1 (3.7)	.334
Breast milk, n (%)	25 (32.9)	11 (45.8)	4 (16.0)	10 (37.0)	.067
RDS, n (%)	25 (32.5%)	17 (68.0)	8 (32.0)	0 (0)	<.004
PDA, n (%)	14 (18.2)	14 (56.0)	0 (0)	0 (0)	<.001
IVH, n (%)	9 (13.6)	7 (28.0)	1 (4.3)	1 (5.6)	0.046
Jaundice, requiring phototherapy, n (%)	19 (24.7)	12 (48.0)	6 (24.0)	1 (3.7)	0.001
Sepsis, culture proven, n (%)	14 (18.2)	11 (44.0)	2 (8.0)	1 (3.7)	<.001
NEC, n (%)	5 (6.5)	5 (20.0)	0 (0)	0 (0)	<.001
FEN, d (mean ± SD)	8.3 ± 13.8	20.4 ± 19.6	2.8 ± 1.3	2.5 ± 2.3	<.001
BPD, n (%)	4 (5.2)	4 (16.0)	0 (0)	0 (0)	.019
Hospital stays, d (mean ± SD)	22.8 (±28.8)	49.1 (38.25)	11.8 (4.27)	8.74 (8.44)	<.001
Mortality, n (%)	2 (2.6)	2 (8.0)	0 (0)	0 (0)	.205

*P* values were obtained by the Kruskal-Wallis tests. Others included preterm labor pain 9, oligo-and polyhydramnios 2, intrauterine growth restriction 2, vaginal bleeding 2, meconium-stained amniotic fluid 3, fetal distress 1.

5′-AS = Apgar score at 5-min after birth, BPD = bronchopulmonary dysplasia, FEN = full enteral nutrition, IVH = intraventricular or intracerebral hemorrhage, NEC = necrotizing enterocolitis stage ≥2, PDA = hemodynamically significant patent ductus arteriosus, PROM = prolonged rupture of membrane, RDS = respiratory distress syndrome.

### 3.2. Plasma MIF concentration and associating clinical factors

**Figure 1.**
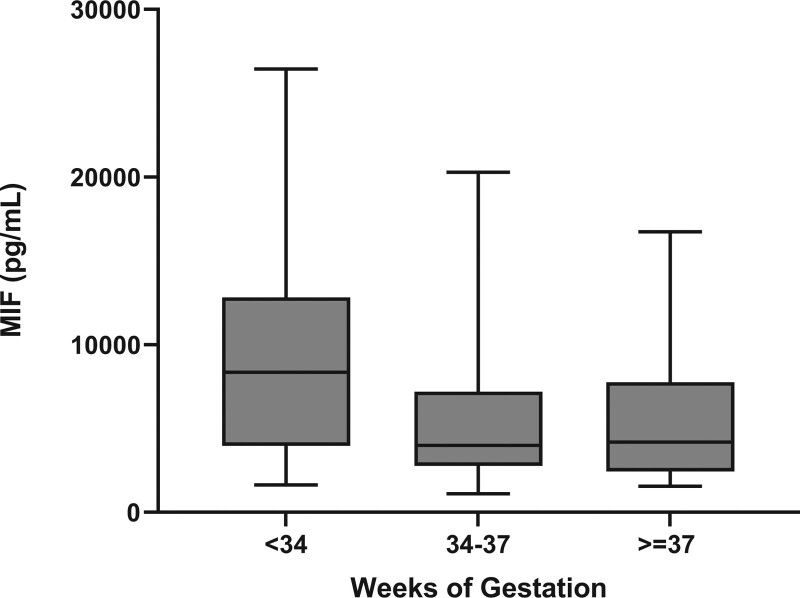
Comparison of the plasma macrophage MIF in the transitional period according to gestational age at birth. Data are presented as means with 95% confidence intervals and statistical difference between the 3 groups was obtained using the Kruskal-Wallis test (*P* = .016). MIF = migration inhibitory factor.

**Figure 2.**
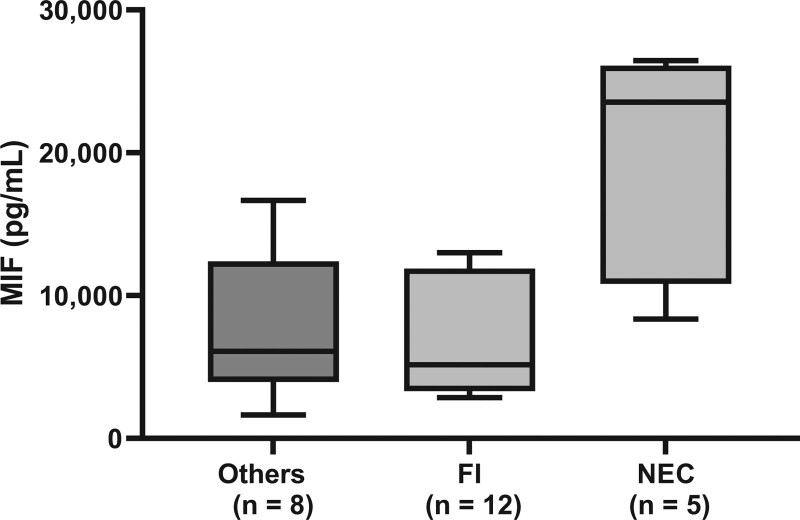
Macrophage migration inhibitory factor in transitional period was associated with development of NEC among 25 preterm neonates born at <34 wk of gestation. Data are represented as mean with 95% confidence interval and statistical difference between the 3 groups was obtained by the Kruskal-Wallis test (*P* = .020). FI = feeding intolerance, NEC = necrotizing enterocolitis, Others = preterm neonates without feeding intolerance nor necrotizing enterocolitis.

The mean plasma MIF concentration was 6934.5 ± 5686.8 pg/mL. Higher MIF concentration was shown in preterm neonates born at <34 weeks of gestation (9849.5 ± 7187.8 pg/mL) than in late preterm (5718.7 ± 4596.4 pg/mL) or term neonates (5361.1 ± 3895.7 pg/mL, *P* = .016, Fig. 1 and Table 4). There was no statistically significant difference between MIF and perinatal factor such as sex (male: 6667.3 ± 5193.7 vs female: 7290.7 ± 6350.5 pg/mL, *P* = .449), delivery method (vaginal delivery: 6536.1 ± 4864.1 vs cesarean section: 7149.6 ± 6121.3 pg/mL, *P* = .357), the presence of obstetric problem (yes: 7190.6 ± 5090.7 vs no: 5782.1 ± 3223.3 pg/mL, *P* = .420), use of prenatal steroid (yes: 7685.6 ± 6206.4 vs no: 6540.3 ± 5463.0 pg/mL, *P* = .157), multiple gestation (yes: 10,849.8 ± 9078.9 vs no: 6480.5 ± 5064.2 pg/mL, *P* = .060), or feeding formula (breast milk: 7522.3 ± 7015.1 vs formula milk: 6320.8 ± 4411.8 pg/mL, *P* = .496, Table 2). Among the 25 preterm neonates born at <34 weeks of gestation, MIF was not statistically significantly different according to RDS (yes: 7494.4 ± 6026.3 vs no: 8073.7 ± 6722.1 pg/mL, *P* = .954), PDA (yes: 10,324 ± 7698.5 vs no: 6796.0 ± 5514.3 pg/mL, *P* = .064), IVH (yes: 10,188.1 ± 8234.3 vs no: 7690.9 ± 6107.1 pg/mL, *P* = .406), neonatal jaundice (yes: 6419.2 ± 4378.9 vs no: 8551.9 ± 7143.5 pg/mL, *P* = .568), sepsis (yes: 9862.3 ± 7605.4 vs no: 7053.9 ± 5750.1 pg/mL, *P* = .152), or BPD (yes: 12,423.7 ± 9615.5 vs no: 7380.7 ± 5942.9 pg/mL, *P* = .132, Table 3). However, plasma MIF was significantly higher in preterm neonates with NEC (19,478.6 ± 8162.4 pg/mL, n = 5) than in those without NEC (7442.4 ± 4553.4 pg/mL, n = 20, *P* = .003, Table 3). In detail, MIF in NEC was significantly different from those in feeding intolerance (7173.7 ± 4203.0 pg/mL, n = 12) and in others (7844.9 ± 5311.2 pg/mL, n = 8, *P* = .020, Fig. 2).

**Table 2:** Comparisons of plasma MIF concentration according to perinatal factors in 77 neonates.

Perinatal factors (n)	MIF concentration (mean ± SD, pg/mL)		*P* value
	Yes	No	
Female(33)	7290.7 ± 6350.5	6667.3 ± 5193.7	.449
Vaginal delivery (27)	6536.1 ± 4864.1	7149.6 ± 6121.3	.357
Obstetric problems (64)	7190.6 ± 5090.7	5782.1 ± 3223.3	.420
Prenatal steroid (27)	7685.6 ± 6206.4	6540.3 ± 5463.0	.157
Multiple gestation (8)	10,849.8 ± 9078.9	6480.5 ± 5064.2	.060
Breast milk (25)	7522.3 ± 7015.1	6320.8 ± 4411.8	.496

*P* value was obtained by Mann–Whitney *U* test. Obstetric problems included prolonged rupture of membrane, maternal hypertensive disorders, maternal gestational diabetes mellitus and others.

MIF = macrophage migration inhibitory factor, SD = standard deviation.

**Table 3:** Comparisons of plasma MIF concentration according to neonatal morbidity among 25 preterm neonates born at <34 wk of gestation.

Neonatal morbidity (n)	MIF concentration (mean ± SD, pg/mL)		*P* value
	Yes	No	
RDS (17)	7494.4 ± 6026.3	8073.7 ± 6722.1	.954
PDA (14)	10,324 ± 7698.5	6796.0 ± 5514.3	.064
IVH (7)	10,188.1 ± 8243.3	7690.9 ± 6107.1	.406
Neonatal jaundice (12)	6419.2 ± 4378.9	8551.9 ± 7143.5	.568
Sepsis (11)	9862.3 ± 7605.4	7053.9 ± 8750.1	.152
NEC (5)	19,478.6 ± 8162.4	7442.2 ± 4553.4	.003
BPD (4)	12,423.7 ± 9615.5	7380.7 ± 5942.9	.132

*P* value was obtained by Mann–Whitney *U* test.

BPD = bronchopulmonary dysplasia, IVH = intraventricular hemorrhage, MIF = macrophage migration inhibitory factor, NEC = necrotizing enterocolitis, PDA = patent ductus arteriosus, RDS = respiratory distress syndrome, SD = standard deviation.

**Table 4:** Comparisons of plasma cytokine concentration between preterm and near or full-term neonates.

Cytokine (mean ± SD, pg/mL)	Total	Preterm neonates		Term neonates	*P* value
		<34	34–37	≥37	
MIF	6934.5 ± 5686.8	9849.5 ± 7187.8	5718.7 ± 4596.4	5361.1 ± 3895.7	.016
IL1β	2.6 ± 15.4	5.3 ± 24.4	1.6 ± 7.3	0 (<0.19)	.889
IL6	43.3 ± 156.4	66.5 ± 184.7	42.9 ± 180.7	11.3 ± 22.1	.588
IL8	171.0 ± 405.7	152.1 ± 214.6	158.7 ± 530.6	215.8 ± 442.6	.018
IL10	35.4 ± 53.5	45.2 ± 53.2	17.7 ± 16.3	44.8 ± 78.3	.072
TNFα	14.9 ± 20.9	16.5 ± 22.2	12.8 ± 20.7	15.6 ± 20.6	.848
IFNγ	10.9 ± 33.9	8.3 ± 18.4	0 (<0.12)	29.6 ± 59.6	.029

*P* value was obtained by the Kruskal-Wallis test. The levels of IL1β in term neonates and IFNγ in preterm born at 34–37 wks of gestation were not detected by enzyme-linked immunosorbent assay.

IFN-γ = interferon γ, IL1β = interleukin 1β, IL6 = interleukin 6, IL8 = interleukin 8, IL10 = interleukin 10, MIF = macrophage migration inhibitory factor, TNFα = tumor necrosis factor-α.

### 3.3. Plasma IL1β, IL6, IL8, IL10, TNF-α, and IFN-γ concentrations

Plasma concentrations of IL1β, IL6, IL8, IL10, TNF-α, and IFN-γ were measured after MIF analysis (Table 4). IL8 was significantly higher in term infants (215.8 ± 442.6 pg/mL) than in preterm (152.1 ± 214.6 pg/mL) or late preterm infants (158.7 ± 530.6 pg/mL, *P* = .018). IL1β and IFN-γ levels were below the detection limit in 96% (55/57) and 86.0% (49/57) of the infants, respectively. The level of under detection of the cytokines set as 0, when performed analysis because under detection meant very low plasma concentration of the cytokine to detect by ELISA. There was no statistical correlation between MIF and the investigated cytokines (IL1β; *r* = −0.185, IL6; *r* = −0.146, *P* = .529, *P* = .423, IL8; *R* = 0.189, *P* = .399, IL10; *R* = 0.347, *P* = .113, TNF-α; *r* = −0.007, *P* = .975, IFN-γ; *r* = −0.258, *P* = .260), when analyzed using non-parametric Spearman correlations.

## 4. Discussion

MIF is a proinflammatory cytokine that plays a significant role in the modulation of diverse immune responses. It has been studied in sepsis, autoimmune diseases, inflammatory bowel diseases, metabolic diseases, and cancers in adults.^[3,10,11]^ However, there are few studies regarding the role of MIF in neonates, and its clinical significance remains unclear. Recently, Roger et al^[2]^ reported that plasma MIF in healthy neonates was 10-fold higher than that in adults, and that MIF obtained from cord blood was higher in full-term neonates than in preterm neonates. However, inconsistent MIF concentrations, such as high levels in cord blood and low levels in amniotic fluid at preterm birth, have been reported previously.^[12,13]^ In this study, the MIF in venous blood from preterm neonates was approximately 2-fold higher than that in late preterm or term neonates (*P* = .016; Fig. 1 and Table 4). Differences in the postnatal day of sample collection, and types of samples might have led to the conflicting results from the previous studies. In other words, previous reports have studied MIF from cord blood and amniotic fluid in the mid-trimester,^[2,4,12,13]^ but we studied MIF from peripheral venous blood of neonates with diverse clinical conditions in the early postnatal period. Since preterm neonates might experience more vulnerable clinical courses in the transitional period than late preterm or term neonates, unstable early clinical conditions such as RDS, PDA, or IVH might affect high MIF concentrations in preterm neonates. However, no statistically significant difference was observed between MIF and the morbidities in transitional period, in this study. And neonatal plasma MIF in the transitional period might not be affected by obstetric problems, use of prenatal steroids, multigestation, mode of delivery, sex, or type of feeding formula in this study. There have been few studies on the role of MIF in respiratory diseases such as RDS and BPD.^[2,14,15]^ Previous studies have suggested that MIF could promote lung development, provoke hypoxia-induced lung injury in mice, or be associated with BPD development.^[2,14–16]^ In this study, we found no significant difference in plasma MIF according to RDS (yes: 7494.4 ± 6026.3 vs no: 8073.7 ± 6722.1 pg/mL, *P* = .954) or BPD (yes: 12,423.7 ± 9615.5 vs no: 7380.7 ± 5942.9 pg/mL, *P* = .132) among the 25 preterm neonates born at <34 weeks of gestation. The conflicting results between the present and previous studies might be caused by different samples, inclusions, and subjects, such as cord blood and tracheal aspirates,^[2,14]^ number of inclusions,^[2,15]^ or humans and mice.^[14,16]^ Since BPD can occur in preterm infants due to multifactorial factors including the arrest of lung development, mechanical trauma, oxygen toxicity, infection, inflammation, and the presence of PDA, some limitations might exist in the prediction of BPD based on MIF levels in the early period of preterm neonates.^[17]^ MIF is a proinflammatory cytokine that has been studied in the pathogenesis of sepsis in adults. However, the role of MIF in neonatal sepsis remains unclear. Roger et al^[4]^ reported that MIF could play a role in sustaining the innate immune response of neonates. In this study, plasma MIF was higher in neonates with sepsis than in those without sepsis, but the difference was not statistically significant (9862.3 ± 7605.4 vs 7053.9 ± 5750.1 pg/mL, *P* = .152). Further studies with larger numbers of inclusions are necessary.

In this study, MIF in preterm neonates who experienced NEC after the transitional period was approximately 2-fold higher than that in preterm neonates without NEC, albeit with a small number of patients with NEC (Fig. 2). In the previous study, up-regulated MIF concentration led to produce IL6 and IL8 from macrophage, and the increment of IL6 and IL8 could aggravate the inflammatory process in NEC.^[18]^ However, Prencipe et al^[19]^ reported functionally relevant polymorphism of -173G/C of MIF promoter, which were associated with higher MIF expression, did not play a role in the development of NEC. Despite the previous contradicted results regarding on the role of MIF in the pathogenesis of NEC, the balance between pro- and anti-inflammatory responses from the multifactorial pathogenetic factors of NEC appears to be fundamental.^[20]^ Recently, there have been studies on the activation of Toll-like receptor (TLR) 4 signaling in the pathogenesis of NEC.^[21–23]^ MIF modulates host immune responses by regulating the expression of TLR4 in response to lipopolysaccharides or by stimulating the production of IL6 and IL8 in severe NEC.^[10,18]^ Based on the activation of the proinflammatory response and TLR4 signaling in NEC, we tentatively suggest that MIF could play a role in the initiation of NEC and that high plasma MIF in preterm neonates during the transitional period could be related to the development of NEC.

We investigated several pro- and anti-inflammatory cytokines with MIF. Among them, IL8 levels were higher in term neonates than in preterm neonates (Table 4). IL8 can play a role in inducing chemotaxis and releasing reactive oxygen metabolites from neutrophils, and has been studied as a biomarker for neonatal sepsis.^[24–26]^ However, IL8 was not significantly different according to sepsis in this study (yes: n = 13, 103.8 ± 158.0 vs no: n = 45, 190.4 ± 452.4 pg/mL, *P* = .948).

This study had several limitations. First, it was a retrospective clinical and exploratory study with a small number of heterogeneous inclusions. Second, we were unable to obtain additional MIF concentrations in preterm neonates with NEC when the disease occurred. Hence, changes in MIF concentrations before and after NEC were not available. Third, since we used remnant blood samples after clinical laboratory tests, there was a possibility of a time lag and an insufficient amount of blood, consequently affecting the concentration of cytokines, including MIF. Despite these limitations, our study had several strengths. We investigated plasma MIF levels in neonates during the transitional period, along with detailed medical reviews unlike the previous reports, and found that preterm neonates had a higher plasma MIF concentration than late preterm or term neonates in the transitional period.^[3,10,12,14,15,18]^ By comparing the concentration of MIF according to neonatal morbidity, we observed that higher MIF levels in the transitional period were significantly associated with the development of NEC in preterm neonates. Further prospective clinical studies on the role of MIF in preterm neonates with NEC are warranted.

## Acknowledgments

The biospecimen and clinical information used in this study were obtained from Gyeongsang National University Hospital, a member of the Korea Biobank Network.

## Author contributions

Ji Sook Park: Conceptualization, funding acquisition and writing-original draft; Jin Su Jun: investigation and methodology; Jae Young Cho: data curation; Jung Sook Yeom: resources and validation; Ji-Hyun Seo: visualization and supervision; Jae Young Lim: formal analysis and supervision; Chan-Hoo Park: methodology and supervision; Hyang Ok Woo: Project administration and supervision; Hee-Shang Youn: Supervision and writing-review and editing.
